# Association of 5-Methylcytosine and 5-Hydroxymethylcytosine with Mitochondrial DNA Content and Clinical and Biochemical Parameters in Hepatocellular Carcinoma

**DOI:** 10.1371/journal.pone.0076967

**Published:** 2013-10-15

**Authors:** Fan Shen, Wei Huang, Jia-Hui Qi, Bi-Feng Yuan, Jing-Tao Huang, Xin Zhou, Yu-Qi Feng, Ying-Juan Liu, Song-Mei Liu

**Affiliations:** 1 Center for Gene Diagnosis, Medical Research Center, Zhongnan Hospital of Wuhan University, Wuhan, Hubei, China; 2 Key Laboratory of Analytical Chemistry for Biology and Medicine (Ministry of Education), Department of Chemistry, Wuhan University, Wuhan, Hubei, China; The University of Hong Kong, China

## Abstract

Increasing epidemiological evidence has indicated that inherited variations of mitochondrial DNA (mtDNA) copy number affect the genetic susceptibility of many malignancies in a tumour-specific manner and that DNA methylation also plays an important role in controlling gene expression during the differentiation and development of hepatocellular carcinoma (HCC). Our previous study demonstrated that HCC tissues showed a lower 5-hydroxymethylcytosine (5-hmC) content when compared to tumour-adjacent tissues, but the relationship among 5-hmC, 5-methylcytosine (5-mC) and mtDNA content in HCC patients is still unknown. This study aimed to clarify the correlation among mtDNA content, 5-mC and 5-hmC by quantitative real-time PCR and liquid chromatography tandem mass spectrometry analysis. We demonstrated that 5-hmC correlated with tumour size [odds ratio (OR) 0.847, 95% confidence interval (CI) 0.746–0.962, *P* = 0.011], and HCC patients with a tumour size ≥5.0 cm showed a lower 5-hmC content and higher levels of fasting plasma aspartate aminotransferase, the ratio of alanine amiotransferase to aspartate aminotransferase, γ-glutamyltransferase, alpha-fetoprotein than those with a tumour size <5 cm (all P<0.05). We further revealed that the mtDNA content of HCC tumour tissues was 225.97(105.42, 430.54) [median (25th Percentile, 75th Percentile)] and was negatively correlated with 5-mC content (*P* = 0.035), but not 5-hmC content, in genomic DNA from HCC tumour tissues.

## Introduction

Mitochondria play an essential role in numerous biological processes such as ATP production, iron and calcium homeostasis, and apoptosis signalling. Human mitochondrial DNA (mtDNA), a 16.6-kb circular double-stranded DNA molecule that lacks introns and protective histones, is susceptible to oxidative DNA damage and exhibits a high mutation rate [1]. Evidence has suggested that mtDNA may also undergo mutations, insertions or deletions in response to oxidative stress in some cancers [2], [3], and mtDNA in tumour cells was found to play an important role in maintaining the malignant phenotype [4].

Variations in mtDNA copy number were also shown to influence the phenotype and the onset of diseases [5] and have been strongly associated with many important human diseases such as diabetes and cancers [6], [7]. A reduction of mtDNA copy number was found in lung cancer patients who were light smokers [8] and in hepatocellular carcinoma (HCC) [9], gastric cancer [10], breast cancer [11] and prostate cancer patients [12]. The reduced mtDNA content found in HCC and invasive breast cancer is significantly associated with the occurrence of somatic point mutations in the D-loop region or with impaired in the mitochondrial biogenesis [13], [14]. In contrast, a progressive increase of the relative mtDNA content was observed in lung cancer patients who were heavy smokers [8], in the carcinogenesis of head and neck cancers [15] and in the progression of oesophageal squamous cell carcinoma [16].

5-Methylcytosine (5-mC), frequently enriched at CpG dinucleotides, is the most important epigenetic marker involved in a variety of cellular processes, including embryogenesis, the regulation of gene expression, genomic imprinting and X-chromosome inactivation [17]. 5-mC can be enzymatically converted to 5-hydroxymethylcytosine (5-hmC) in mammalian DNA by ten-eleven translocation (TET) [18]. Epigenetic changes in methylation patterns are largely implicated in cancer development [19]–[21]. In a broad panel of cancers, including HCC, lower global DNA methylation was observed in tumour tissues when compared with normal tissue counterparts [22]–[24]. Experimentally induced hypomethylation can lead to cancer development in animal studies, indicating that global DNA hypomethylation plays a causal role in cancer development [25]–[27], and lower levels of global methylation were reported in leukocyte DNA from colorectal adenoma patients [28], [29]. The demethylation of the D-loop also played a key role in regulating ND2 (a subunit of NADH) expression during the initiation and/or progression of colorectal cancer [30]. We have demonstrated that HCC tumour tissues have a lower 5-hmC content when compared to tumour-adjacent tissues and that 5-hmC content is highly correlated with tumour stages [21].

However, the correlation among mtDNA content, 5-mC, and 5-hmC in HCC patients has not been explored. In recent years, mtDNA content has been measured frequently as the ratio of mtDNA to nuclear DNA using quantitative real-time PCR (qPCR), and the results are often termed as the mtDNA copy number or mtDNA content [7]. Hence, the present study aimed to investigate the correlation among mtDNA content, 5-mC, and 5-hmC from HCC tumour tissues by qPCR and liquid chromatography tandem mass spectrometry system and to examine whether mtDNA content, 5-mC and 5-hmC are correlated with clinico-pathological parameters.

## Materials and Methods

### Study Population, Specimens and Epidemiologic Data

This prospective study was approved by the ethics committee of Zhongnan Hospital of Wuhan University, and written informed consent was obtained from all patients prior to their enrolment in the study. A total of 144 HCC patients (117 males and 27 females, age 48.8±12.3 years, range 18–80 years) were enrolled from June 2005 to January 2012 at Zhongnan Hospital of Wuhan University without any restriction on age, gender or tumour stage (involved tumour-nodes-metastasis (TNM) stage I (n = 92), Stage II (n = 14), Stage III (n = 19), Stage IV (n = 16) and unknown stage (n = 3) cancer). None of the cases had a previous history of other cancers or treatments. The demographic and personal data of all participants were collected using a standardised epidemiological questionnaire, including age, gender, smoking, drinking status, family history of cancer, and history of hepatitis virus infection. The patients who had smoked ≥100 cigarettes in their lifetime were defined as smokers, and those who had consumed at least 80 g/day alcohol for more than 1 year were considered to be drinkers. All patients were pathologically confirmed and underwent a liver resection, and 144 formalin-fixed, paraffin-embedded HCC tumour tissue samples were collected. Tumour sizes ranged from 2.8 to 20.0 cm, with a mean size of 8.0±4.6 cm. A total of 4 mL fasting blood sample was obtained from each participant for the detection of clinico-pathological parameters. Among these patients, 116 were positive for serum hepatitis B surface antigen, and 28 were positive for serum anti-hepatitis B virus antibody. Table 1 and Table 2 list the baseline characteristics and preoperative clinico-pathological parameters of the HCC patients.

**Table 1:** Baseline characteristics of HCC patients.

Baseline variables	HCC patient cases (n, %)
Gender	
male	117(81.3%)
female	27(18.8%)
Smoking	
Yes	55(38.2%)
No	89(61.8%)
Drinking status	
Yes	42(29.2%)
No	102(70.8%)
Pugh-Child’s classification	
A	116(87.9%)
B	16(12.1%)
AFP (ng/ml)	
<20	28(21.4%)
≥20	93(78.6%)
Tumour size (cm)	
<5.0	36(25.0%)
≥5.0	108(75.0%)
HBV infection	
Yes	116(80.6%)
No	28(19.4%)
Cirrhosis	
Yes	45(31.3%)
No	99(68.7%)
Liver inflammation	
without HBV or HCV infection	20(13.9%)
mild hepatitis	33(22.9%)
moderate hepatitis	31(21.5%)
severe hepatitis	26(18.1%)
liver failure	34(23.6%)

**Table 2:** Clinico-pathological parameter values of HCC patients.

Clinico-pathological parameters	Values^a^	Reference Intervals
Alanine amiotransferase, ALT (U/L)	37(29, 75)	0–46
aspartate aminotransferase, AST (U/L)	44(31, 71)	0–46
ALT/AST	1.07(0.83, 1.33)	0.2–2
Total bilirubin, TBILI (µmol/L)	18.7(14.2, 32.5)	0–25
Direct bilirubin, DBILI (µmol/L)	4.0(3.3, 7.1)	0–7
Indirect bilirubin, IBILI (µmol/L)	14.1(10.0, 17.7)	1.5–18
Total protein, Tp (g/L)	67.4±8.9	60–80
Albumin, Alb (g/L)	39.8±5.8	35–55
Globulin, Glb (g/L)	27.3±5.2	20–30
Alb/Glb	1.50(1.29, 1.68)	1.5–2.5
γ-glutamyltransferase, GGT (U/L)	55(37, 105)	5–55
Alkaline phosphatase, ALP (U/L)	95(71, 131)	35–134
5-Nucleotidase, 5′NT (U/L)	3(2, 6)	0–10
Total biliary acid, TBA (µmol/L)	7.2(3.7, 14.5)	0–15
Cholinesterase, CHE (U/L)	6117±2273	3000–10500
Pre-albumin, PALB (g/L)	0.12(0.07, 0.18)	0.1–0.4
Fasting plasma glucose, FBG (mmol/L)	5.0(4.5, 6.3)	3.9–6.2
Blood urea nitrogen, BUN (mmol/L)	4.9(3.8, 6.0)	1.7–7.2
Creatinine, Cr (µmol/L)	76.0(68.5, 83.9)	45–117
Uric acid, UA (µmol/L)	244.3±83.4	119–417
Retinol-binding protein, RBP (mg/L)	26.3±12.8	15–70
Cystatin C, Cys C (mg/L)	1.0(0.9, 1.2)	0–1.2
Carcinoembryonic antigen, CEA (ng/ml)	2.0(1.3, 3.4)	0–5
Alpha-fetoprotein, AFP (ng/ml)	200(20.3, 1000)	0–20
Ferritin (ng/ml)	267.4(107.2, 424.6)	0–322
Cancer antigen 125, CA125 (KU/L)	18.6(10.2, 36.4)	0–35
Cancer antigen 153, CA153 (KU/L)	10.3(7.3, 13.0)	0–35
Cancer antigen 199, CA199 (KU/L)	9.3(5.0, 23.3)	0–35
Total prostate specific antigen, Total PSA (ng/ml)	0.66(0.36, 0.94)	0–5
Free prostate specific antigen, Free PSA (ng/ml)	0.17(0.11, 0.30)	0–1
Prothrombin time, PT (sec)	12.1(11.3, 13.0)	10.5–13.5
PT% (%)	88.6(80.8, 101.1)	80–130
International standard ratio, INR	1.05(0.98, 1.13)	0.85–1.15
D-fibrinogen, DFbg (g/L)	2.8(2.2, 3.4)	2–4
Thrombin time, TT (sec)	14.4(13.3, 15.3)	11–14

a Data were expressed as median (25th Percentile, 75th Percentile) or Mean ± SD.

### DNA Extraction

Total genomic DNA was extracted using a commercial kit from TaKaRa DEXPAT Easy (TaKaRa, Dalian, China) according to the manufacturer’s instructions. DNA was quantified with a DU®530 spectrophotometer (Beckman Coulter, Fullerton, CA, USA). The extracted DNA samples were frozen at −20°C until use.

### Determination of mtDNA Content by qPCR

mtDNA content was estimated by amplification from the total genomic DNA. Primer sequences for mtDNA tRNALeu^(UUR)^ and nuclear DNA β2-microglobulin (β2M) have been described previously [31]. qPCR was performed on the Mx3000P™ Real-time PCR System (Stratagene, USA). The 25 µL reaction mixture contained 10 ng of genomic DNA, 12.5 µL of 2×Platinum SYBR Green qPCR SuperMix-UDG with Rox (Invitrogen, Shanghai, China), 25 µM of forward and reverse primers, and 6.5 µL ddH_2_O. The thermal cycling conditions were 95°C for 1 min, followed by 40 cycles of 95°C for 30 s, 62°C for 30 s, and 72°C for 30 s with signal acquisition. After amplification, the dissociation curve was provided to confirm the quality of the qPCR reaction. A duplicate control without DNA template was included in each run to test the buffers and solutions for contamination and to assess the formation of any primer-dimers.

The standard curves for tRNALeu^(UUR)^ and β2M were linear in the tested ranges (R^2^ = 0.991 for tRNALeu^(UUR)^, R^2^ = 0.996 for β2M). All C_T_ values of unknown samples fell within the linear range. The slopes of the standard curves for tRNALeu^(UUR)^ and β2M were −3.348 and −3.027, respectively. High amplification efficiencies of 98.9% and 114.0% were determined for both tRNALeu^(UUR)^ and β2M in the range investigated [32] and were used for the quantification of unknown samples.

mtDNA content was calculated using the following formula: 2×2 (ΔC_T_), where ΔC_T_ is the difference of C_T_ values between the β2M and the tRNALeu^(UUR)^ [31], and the result was expressed as the median (25th percentile, 75th percentile).

### Measurement of 5-mC and 5-hmC

The quantification of 5-mC and 5-hmC in genomic DNA was performed according to our previous study [21]. Briefly, 50 ng of genomic DNA was dissolved in 13 µL ddH_2_O, denatured at 95°C for 5 min, and chilled on ice for 2 min. Then, 1.5 µL of 10×S1 nuclease buffer and 100 units (0.5 µL) of S1 nuclease (TaKaRa, Dalian, China) were subsequently added, and the mixture (15 µL) was incubated at 37°C for 4 h. After adding 6 µL of 10×alkaline phosphatase buffer, 0.002 units (2 µL) of venom phosphodiesterase I (Sigma, St. Louis, MO), 15 units (0.5 µL) of alkaline phosphatase (TaKaRa, Dalian, China) and 36.5 µL ddH_2_O, the mixture (60 µL) was incubated at 37°C for an additional 2 h. Next, the solution was diluted with 440 µL ddH_2_O and extracted with 500 µL phenol/chloroform (1/1, v/v) once and with 500 µL chloroform twice. The lyophilised nucleoside extracts were dissolved in 200 µL acetonitrile/H_2_O (99/1, v/v) and centrifuged at 10,000 g for 5 min. The supernatant was then analysed by liquid chromatography tandem mass spectrometry (Bruker Daltonics, Bremen, Germany). Hydrophilic poly(*N*-acryloyltris(hydroxymethyl)aminomethane*-co-*pentaerythritol triacrylate, NAHAM*-co-*PETA) monolithic capillary (50 cm long, 100 µm inner diameter, 360 µm outer diameter) (Weltech, Wuhan, China) was used for the separation of target analytes. The parameters for the analysis were as follows: dry gas temperature, 150°C; ISCID energy, 15 eV; hexapole RF, 205 Vpp; quadrupole ion energy, 20 eV; collision energy, 12 eV; collision RF, 150 Vpp.

### Statistical Analysis

The statistical package SPSS version 17.0 (SPSS Inc., Chicago, USA) was used for all statistical analyses. Normally distributed data were expressed as the mean ± SD, and Student’s t-test or one-way ANOVA was used to assess the differences between continuous variables. Skewed data were described by the median and interquartile range, and group comparison was performed with a non-parametric analysis. Pearson’s correlation analysis was applied to evaluate the correlation between mtDNA content and the clinico-pathological parameters. The associations among mtDNA content, 5-mC, 5-hmC, tumour size, and HCC risk factors were estimated by odds ratios (ORs) and corresponding 95% confidence intervals (CIs) using unconditional logistic regression analysis, with adjustment for possible confounders. All statistical tests were two-sided, and the level of statistical significance was set at *P*<0.05.

## Results

### Correlations among mtDNA Content, 5-mC, and 5-hmC

The mtDNA content was 225.97(105.42, 430.54) [median (25th Percentile, 75th Percentile)] in 144 HCC tumour tissues. The average contents of 5-mC and 5-hmC were 5.59±0.84% and 0.38±0.20%, respectively, in 110 HCC tumour tissues. Pearson’s correlation coefficient revealed a negative correlation between the mtDNA content and 5-mC (r = −0.201, *P* = 0.035). However, there was no correlation between mtDNA content and 5-hmC (*P* = 0.244).

### Correlations among mtDNA Content, 5-mC, 5-hmC, and Clinico-pathological Parameters

We further evaluated the correlations among mtDNA content, 5-mC, 5-hmC, and clinico-pathological parameters by Pearson’s correlation analysis. Our data suggested that the mtDNA content was only related to the DFbg level (r = 0.213, *P* = 0.027); 5-mC content significantly correlated with the values for DBILI (r = −0.224, *P* = 0.021), Alb/Glb (r = 0.195, *P* = 0.045) and GGT (r = −0.223, *P* = 0.022), while 5-hmC content was only associated with TBA level (r = 0.739, *P*<0.001).

### Correlations among Tumour Size, HCC Risk Factors and mtDNA Content, 5-mC, and 5-hmC

To examine the associations among mtDNA content, 5-mC, 5-hmC, tumour size, and HCC risk factors (age, gender, smoking, drinking status, and HBV infection), we performed unconditional logistic regression analysis, with adjustment for possible confounders based on the median mtDNA content or the average 5-mC and 5-hmC contents. As shown in Table 3, mtDNA content was associated with age [OR 0.968, 95% CI (0.938–0.999), *P* = 0.046]; 5-hmC was associated with tumour size [OR 0.847, 95% CI (0.746–0.962), *P* = 0.011]; 5-mC may be related to tumour size [OR 0.898, 95% CI (0.807, 1.000), *P* = 0.05]. There were no correlations among gender, smoking, drinking status, HBV infection, mtDNA content, 5-mC, and 5-hmC (all *P*>0.05).

**Table 3:** Correlations among tumour size, HCC risk factors and mtDNA content, 5-mC, and 5-hmC.

	mtDNA content^a^				
	<225.97 (n = 72)	≥225.97 (n = 72)	P value^b^	P value^c^	OR (95% CI)^c^
Age (years), mean±SD	50.5±11.0	47.1±13.2	0.098	0.046	0.968 (0.938,0.999)
Gender, n (%)			0.522	0.111	2.279 (0.828,6.274)
male	57 (79.2)	60 (83.3)			
female	15 (20.8)	12 (16.7)			
Smoking, n (%)			0.123	0.440	1.469 (0.554,3.891)
Yes	32 (44.4)	23 (31.9)			
No	40 (55.6)	49 (68.1)			
Drinking status, n (%)			0.271	0.879	0.922 (0.326,1.091)
Yes	24 (33.3)	18 (25.0)			
No	48 (66.7)	54 (75.0)			
Tumour size (cm), mean±SD	7.9±4.9	8.1±4.2	0.854	0.852	1.008 (0.930,1.091)
HBV infection, n (%)			0.092	0.375	0.642 (0.241,1.708)
Yes	54 (75.0)	62 (86.1)			
No	18 (25.0)	10 (13.9)			
	**5-mC content** d				
	**<5.59% (n = 55)**	**≥5.59% (n = 55)**	**P value** b	**P value** c	**OR (95% CI)** c
Age (years), mean±SD	49.6±14.2	48.3±10.7	0.603	0.606	0.990 (0.954,1.028)
Gender, n (%)			0.429	0.373	1.814 (0.490,6.722)
male	45 (81.8)	48 (87.3)			
female	10 (18.2)	7 (12.7)			
Smoking, n (%)			0.006	0.073	0.352 (0.113,1.100)
Yes	13 (23.6)	27 (49.1)			
No	42 (76.4)	28 (50.9)			
Drinking status, n (%)			0.145	0.842	0.884 (0.262,2.978)
Yes	13 (23.6)	20 (36.4)			
No	42 (76.4)	35 (63.6)			
Tumour size (cm), mean±SD	8.5±4.4	6.9±4.2	0.074	0.050	0.898 (0.807,1.000)
HBV infection, n (%)			0.101	0.278	0.516 (0.156,1.706)
Yes	40 (72.7)	47 (85.5)			
No	15 (27.3)	8 (14.5)			
	**5-hmC content** d				
	**<0.38% (n = 72)**	**≥0.38% (n = 38)**	**P value** b	**P value** c	**OR (95% CI)** c
Age (years), mean±SD	48.9±12.9	49.3±11.9	0.877	0.386	0.983 (0.946,1.022)
Gender, n (%)			0.944	0.717	1.283 (0.334,4.936)
male	61 (84.7)	32 (84.2)			
female	11 (15.3)	6 (15.8)			
Smoking, n (%)			0.622	0.807	1.156 (0.363,3.683)
Yes	25 (34.7)	15 (39.5)			
No	47 (65.3)	23 (60.1)			
Drinking status, n (%)			0.484	0.225	0.469 (0.138,1.594)
Yes	20 (27.8)	13 (34.2)			
No	52 (72.2)	25 (65.8)			
Tumour size (cm), mean±SD	8.5±4.6	6.3±3.6	0.016	0.011	0.847 (0.746,0.962)
HBV infection, n (%)			0.311	0.083	2.879 (0.870,9.524)
Yes	59 (81.9)	28 (73.7)			
No	13 (18.1)	10 (26.3)			

Notes: ^a^based on the median mtDNA content (225.97);

b p values derived from the chi-square test or Student’s t-test;

c ORs (95% CIs) and p values derived from logistic regression after the variables were adjusted.

d based on the average 5-mC (5.59%) or 5-hmC (0.38%) content.

### Comparative Analysis of mtDNA Content, 5-mC, 5-hmC, and Clinico-pathological Parameters based on Tumour Size, Cirrhosis, and Liver Inflammation

**Figure 1:**
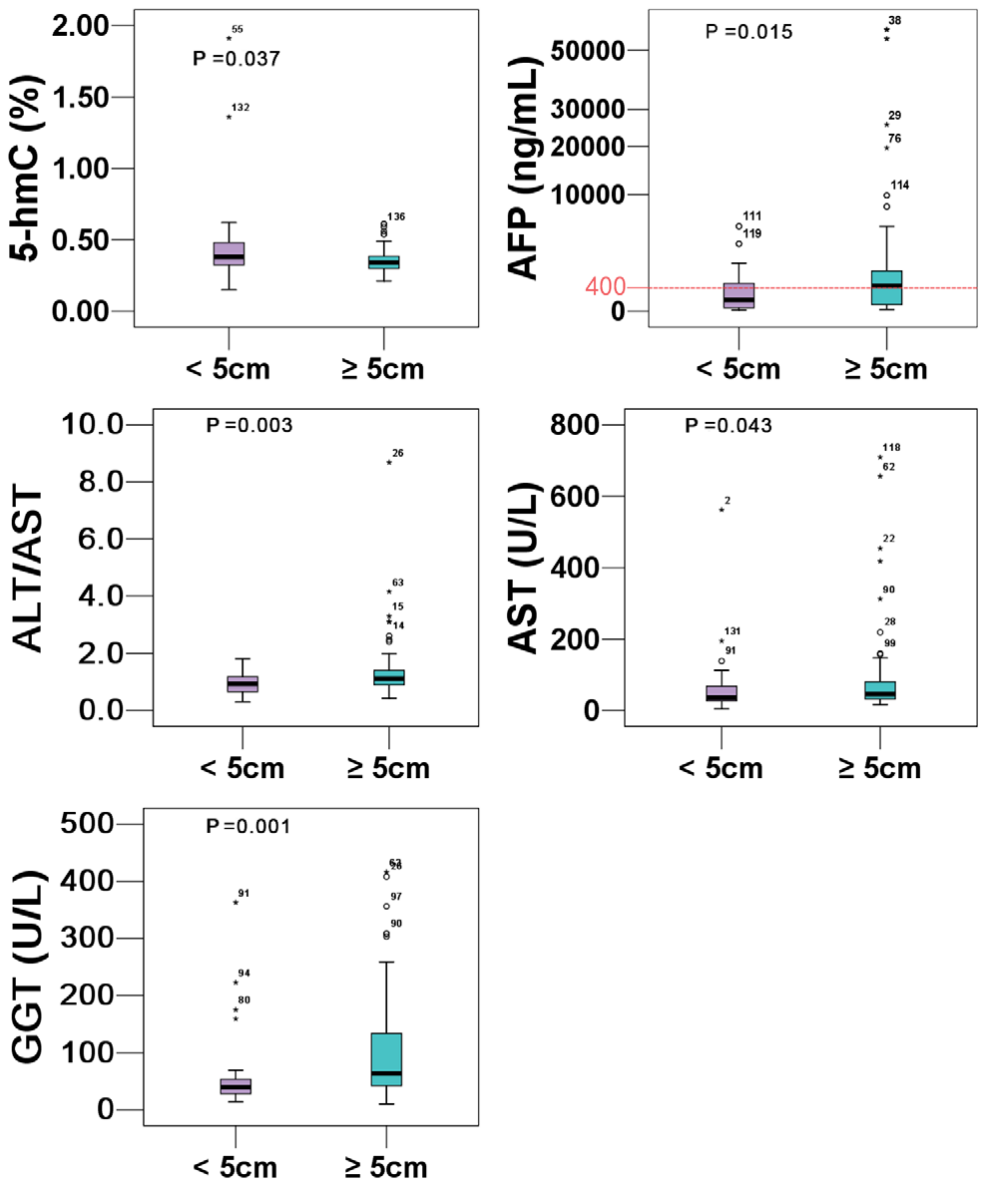
Comparisons of 5-hmC and clinico-pathological parameters in HCC based on tumour size.

To investigate the possible effects of tumour size on mtDNA content, 5-mC, 5-hmC and clinico-pathological parameters in HCC, we divided the HCC patients into two groups according to the tumour size: the <5.0 cm group (n = 36) and the ≥5.0 cm group (n = 108), then a non-parametric Mann-Whitney test and Student’s t-test were used for the group comparison of skewed data and normally distributed data, respectively. As illustrated in Fig. 1, the ≥5.0 cm group showed lower 5-hmC content [median (25th Percentile, 75th Percentile): 0.34 (0.30, 0.39) vs. 0.38 (0.32, 0.49), *P* = 0.037] and higher levels of fasting plasma AST [47.0 (34.0, 85.0) vs. 36.0 (27.0, 67.8), *P* = 0.043], ALT/AST [1.11 (0.89, 1.49) vs. 0.94 (0.65, 1.18), *P* = 0.003], GGT [65.0 (42.0, 132.8) vs. 39.5 (27.5, 55.0), *P* = 0.001], and AFP [517.1 (30.6, 1083.0) vs. 90.0 (8.6, 386.6), *P* = 0.015]. Apart from the above described parameters, there was no significant difference between the two groups (all *P*>0.05).

To identify the effects of cirrhosis on mtDNA content, 5-mC, 5-hmC and clinico-pathological parameters, we categorised the HCC patients into two groups according to the presence or absence of cirrhosis. A non-parametric Mann-Whitney test indicated that the cirrhosis group had an obviously longer TT [median (25th Percentile, 75th Percentile):14.7 (14.0, 15.5) vs. 14.0 (12.9, 14.8), *P* = 0.019]. The non-parametric Two-Sample Kolmogorov-Smirnov test revealed that the mtDNA content in the cirrhosis group was significantly higher than in the non- cirrhosis group [272.5 (112.2, 487.9) when compared to 155.5 (94.2, 365.2), *P* = 0.035]. No difference was observed in 5-mC or 5-hmC content between the two groups (all *P*>0.05).

**Figure 2:**
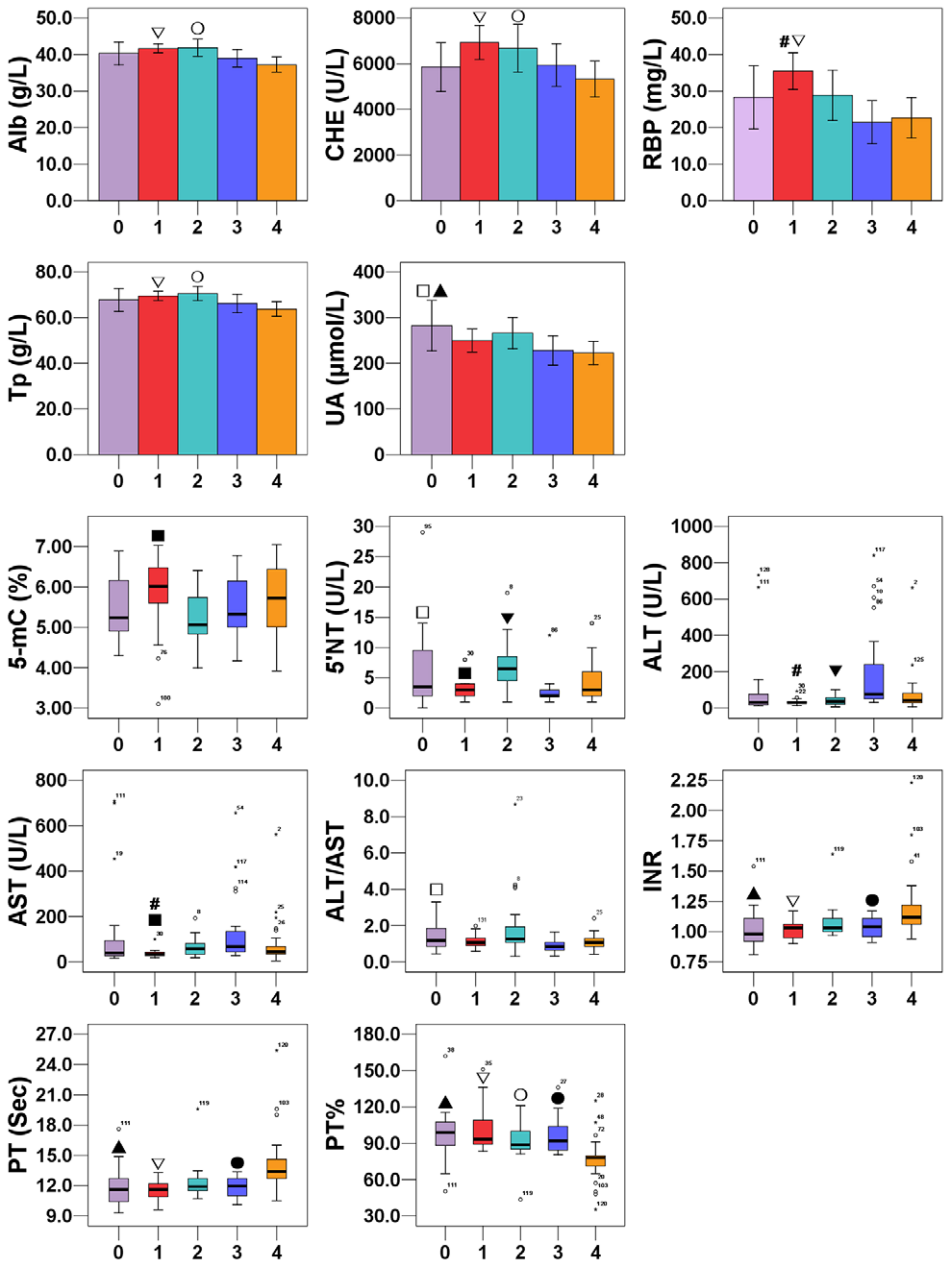
Comparisons of 5-mC and clinico-pathological parameters in HCC based on liver inflammation. 0 = the group without HBV or HCV infection (n = 20), 1 = the mild hepatitis group (n = 33), 2 = the moderate hepatitis group (n = 31), 3 = the severe hepatitis group (n = 26), 4 = the liver failure group (n = 34). Bar charts and Boxplots indicate the results of One-way ANOVA and K-Independent non-parametric analysis, respectively. □: Group-0 vs. Group-3, ▴: Group-0 vs. Group-4, ▪: Group-1 vs. Group-2, #: Group-1 vs. Group-3, ∇: Group-1 vs. Group-4, ▾: Group-2 vs. Group-3, ○: Group-2 vs. Group-4, •: Group-3 vs. Group-4.

Next, the subjects were divided into 5 groups according to their stage of liver inflammation: without HBV or HCV infection (n = 20), mild hepatitis (n = 33), moderate hepatitis (n = 31), severe hepatitis (n = 26) and liver failure (n = 34). One-way ANOVA and K-Independent non-parametric analysis demonstrated that there were differences among the 5 groups in Alb, CHE, UA, RBP, 5-mC, ALT, AST, ALT/AST, Tp, 5′NT, PT, PT% and INR. Thus, LSD and Games-Howell multiple comparisons were used to further evaluate these differences. As shown in Fig. 2: compared to the group without HBV or HCV infection, the severe hepatitis group showed lower ALT/AST, 5′NT and UA levels; the liver failure group had lower UA and PT% and higher PT and INR levels (all *P*<0.05). Compared with the mild hepatitis group, the moderate hepatitis group had increased AST and 5′NT and decreased 5-mC levels; the severe hepatitis group had elevated ALT and AST and reduced RBP levels; the liver failure group had higher PT and INR levels and lower PT%, Tp, Alb, CHE, and RBP levels (all *P*<0.05). Compared with the moderate hepatitis group, the severe hepatitis group had higher ALT and lower 5′NT levels; the liver failure group showed lower Tp, Alb, CHE, and PT% levels (all *P*<0.05). Finally, the severe hepatitis group had higher PT% and lower PT and INR levels than the liver failure group (all *P*<0.05).

## Discussion

Due to the alterations of mtDNA in various tumours including hepatoma and meningioma, as well as the insertion of mtDNA sequences into the nuclear genome [33]–[35], cancers have been described as a ‘mitochondriopathy’ [36]. HCC is a highly lethal malignant tumour with an increasing incidence [37]. Previous studies demonstrated that mtDNA, 5-mC and 5-hmC abnormalities play certain roles in the pathological process of HCC [9], [21], [24]. In the present study, we quantified mtDNA content, 5-mC, and 5-hmC in genomic DNA from HCC tumour tissues by qPCR and liquid chromatography tandem mass spectrometry analysis.

The data here presented demonstrated that 5-mC content in genomic DNA from HCC tumour tissues was negatively correlated with mtDNA content. Our results confirmed that the mitochondrial DNA copy number was regulated by DNA methylation. It is well-documented that mtDNA replication and integrity maintenance are controlled by the nDNA [38]; both mitochondrial transcription factor A (TFAM) and the nuclear-encoded polymerase gamma (POLG), which is composed of one POLGA catalytic subunit and two POLGB accessory subunits, are essential for mtDNA replication [38], [39]. More recently, Kelly et al observed a negative correlation between intragenic DNA methylation and the levels of POLGA expression, which were negatively correlated with the mtDNA copy number as determined by bisulphite sequencing, restriction enzyme digest real-time PCR and methylated DNA immunoprecipitation with real-time PCR [40]. The expression of TFAM is also associated with embryogenesis and the regulation of the mtDNA copy number [39]. Analysis of the TFAM promoter has shown that DNA methylation within a nuclear respiratory factor-1 binding site may inhibit TFAM expression and decrease mtDNA biogenesis [41]. Likewise, the promoter hypermethylation of transcriptional peroxisome proliferator-activated receptor gamma coactivator 1-α (PGC-1α), an important factor in mitochondrial biogenesis and energy metabolism, was negatively correlated with PGC-1α mRNA and mtDNA content in type 2 diabetic patients [42]. Moreover, mitochondria can influence cytosine methylation levels in the nucleus by modulating the flux of one-carbon units for the generation of S-adenosylmethionine, which is a universal substrate of cytosine methylation and plays an essential role in the epigenetic regulation of nuclear gene expression [43], [44]. These findings indicate an epigenetic mechanism for the regulation of mtDNA replication.

There was no correlation between 5-hmC and mtDNA content observed in our study. In the nucleus, 5-hmC is generated from 5-mC by oxidation with TET family oxygenases [45]. To date, it is still unclear whether the TET family proteins are present in mitochondria and whether the TET family proteins or loci contain recognisable mitochondrial targeting sequences [46]. Although Shock *et al* demonstrated that both 5-hmC and 5-mC are present in mammalian mtDNA [43], both are present at very low levels, especially 5-hmC [18], [40]. Another possible reason for no correlation between 5-hmC and mtDNA content is that we did not directly quantify the 5-hmC content of the mitochondrial genome.

Our results confirmed that age was correlated with mtDNA content in HCC tumour tissues [OR 0.968, 95% CI (0.938–0.999), *P* = 0.046]. A recent study reported that mtDNA content increased with age in muscle tissue, decreased with age in peripheral blood leukocyte, and appeared to be unaffected by age in liver tissue [47]. Zhao *et al* showed that there was no significant difference in mtDNA content between younger (<48 years) and older (≥48 years) HCC patients [48]. Although the mechanism behind this decrease in mtDNA content with age in HCC tumour tissues is still not clear, the accumulated genetic alterations weaken mitochondrial functions and DNA repair systems in older population, and this may play an important role in the alteration of mtDNA content. Similar results were reported by Yamada *et al* [9], with no significant correlation between mtDNA content and gender, smoking, drinking status, hepatitis virus, tumour size, clinico-pathological parameters, Pugh-Child’s classification and TNM stage.

It is worth noting that 5-hmC was associated with tumour size [OR 0.847, 95% CI (0.746–0.962), *P* = 0.011] and that HCC patients with a tumour size ≥5.0 cm showed a lower 5-hmC content and higher levels of serum AST, ALT/AST, GGT and AFP. This finding is in accordance with previous works demonstrating that serum ALT, AST, AFP [49] and GGT [50] levels are effective predictors of HCC risk. Likewise, Liu *et al* recently reported that the decreased 5-hmC in HCC was associated with tumour size and AFP level [51]. Also, our previous study showed that 5-hmC content highly correlated with tumour stage (TNM and Barcelona Clinic Liver Cancer) [21]. Additionally, the results of our preliminary correlation analysis showed that 5-mC content significantly correlated with DBILI, Alb/Glb and GGT, while 5-hmC only correlated with TBA. This finding indicates that both 5-mC and 5-hmC are associated with the damaged liver function. Therefore, 5-hmC and 5-mC combined with routinely tested liver functional parameters may serve as a potential biomarker for HCC early detection.

Subsequently, we performed subgroup analysis according to the stages of liver inflammation and fibrosis because the molecular process in hepatocarcinogenesis can differ greatly based on aetiology. Our data suggested that 5-mC and some clinico-pathological parameters (ALT, AST, ALT/AST, Tp, Alb, 5′NT, CHE, UA, RBP, AFP, PT, PT%, INR, DFbg and TT) were affected by cirrhosis or liver inflammation (Fig. 2). As expected, these clinico-pathological parameters reflected the damaged liver function and the destruction of hepatocellular carcinoma tissues. Our data were consistent with an earlier study showing that mtDNA content was correlated with cirrhosis [52], and that HCC patients with cirrhosis had elevated mtDNA content. Paradoxically, we have found that the moderate hepatitis group showed lower 5-mC content than the mild hepatitis group, while no differences were found between other groups. To some extent, this finding suggests that 5-mC is not highly associated with the stage of hepatitis in HCC patients.

Taken together, our data provide the first evidence that mtDNA content is negatively correlated with 5-mC content, but not 5-hmC content in genomic DNA from HCC tumour tissues. The linkages among mtDNA content, 5-mC, and 5-hmC in the genomic DNA of HCC tumour tissues will provide an important new insight on the pathogenesis of HCC.
